# Reducing postpartum weight retention – a pilot trial in primary health care

**DOI:** 10.1186/1475-2891-6-21

**Published:** 2007-09-10

**Authors:** Tarja I Kinnunen, Matti Pasanen, Minna Aittasalo, Mikael Fogelholm, Elisabete Weiderpass, Riitta Luoto

**Affiliations:** 1 UKK Institute for Health Promotion Research, PO Box 30, 33501 Tampere, Finland; 2 Research Unit, Pirkanmaa Hospital District, Tampere, Finland; 3 Department of Medical Epidemiology and Biostatistics, Karolinska Institutet, Stockholm, Sweden; 4 Department of Etiological Research, Institute of Population-Based Cancer Research, Cancer Registry of Norway, Oslo, Norway; 5 Department of Genetical Epidemiology, Folkhalsan Research Center, Samfundet Folkhälsan, Helsinki, Finland; 6 Tampere School of Public Health, the University of Tampere, Tampere, Finland

## Abstract

**Background:**

Postpartum weight retention may contribute to the development of obesity. We studied whether individual counselling on diet and physical activity from 2 to 10 months postpartum has positive effects on diet and leisure time physical activity and increases the proportion of primiparas returning to their pre-pregnancy weight.

**Methods:**

A controlled trial including ninety-two postpartum primiparas was conducted in three intervention and three control child health clinics in primary health care in Finland. The intervention included individual counselling on diet and physical activity during five routine visits to a public health nurse; the controls received the usual care.

**Results:**

In total, 50% of the intervention group and 30% of the control group returned to their pre-pregnancy weight (weight retention ≤ 0 kg) by 10 months postpartum (p = 0.06). The confounder-adjusted odds ratio for returning to pre-pregnancy weight was 3.89 (95% CI 1.16–13.04, p = 0.028) for the intervention group compared with the controls. The mean proportion of high-fibre bread (of total weekly amount of bread) increased by 16.1% (95% CI 4.3–27.9) by 10 months postpartum in the intervention group compared with the controls when adjusted for confounders (p = 0.008). No significant differences were observed in changes in leisure time physical activity between the groups.

**Conclusion:**

The intervention increased the proportion of primiparas returning to pre-pregnancy weight and the proportion of high-fibre bread in their diet. Larger randomized controlled trials are needed to show whether counselling can improve dietary and leisure time physical activity habits in postpartum women and also to confirm the results concerning the effect on reducing postpartum weight retention.

**Trial registration:**

Current Controlled Trials ISRCTN21512277

## Background

Obesity is a growing problem which also increases the burden of several diseases such as type 2 diabetes, cardiovascular disease and certain cancers [1]. In Finland, 41% of women aged 15–64 years were overweight or obese (body mass index, BMI ≥ 25 kg/m^2^) in 2006, but the data is based on self-reported information [2]. For some women, pregnancy is a triggering factor for long-term overweight and obesity [3]. Postpartum weight retention is usually highly variable and a subgroup of women retains large amounts of weight after pregnancy. In some studies, up to 20 percent of women have retained at least 5 kg by 6–18 months postpartum [4]. The average postpartum weight retention varies from 0.5 kg to 3 kg in different study populations [5].

Excessive gestational weight gain is the primary risk factor for retaining weight in the postpartum period [4-6]. Other factors associated with an increased risk of high postpartum weight retention include high pre-pregnancy BMI, primiparity, short duration of breastfeeding, stopping smoking, high energy intake and low physical activity, although these associations have not been found in all studies [4,7]. Only few studies have assessed the influence of diet and physical activity on postpartum weight change. Higher or increased energy intake and lower physical activity were associated with higher postpartum weight retention in some studies [8-10], but not in all [11].

Relatively few weight loss interventions have been conducted among postpartum women. Only two of these studies aimed primarily to reduce postpartum weight retention [12,13], while the other studies aimed to investigate the effect of weight loss on lactation or child growth [14-16]. In most of these studies, the intervention consisted of a prescribed diet and an exercise programme. In four studies, the women in the intervention group lost more weight than the women in the control group [12,13,15,16], but all these studies were small and/or had a high dropout-rate. More information is needed on the effect of behavioural interventions to prevent weight retention in this group of women [5].

The aim of the study was to investigate whether individual counselling on diet and physical activity after pregnancy has positive effects on diet and leisure time physical activity (LTPA) and increases the proportion of primiparas who return to their pre-pregnancy weight by 10 months postpartum. This study is a part of a pilot study testing the feasibility of the study protocol for a larger study also including pregnant women [17].

## Methods

### Setting and general study design

The postpartum women were recruited through the child health care system, which is available to all families with children in every municipality in Finland and is funded by public tax revenue. Almost all (98%) children attend these public child health clinics (CC) for regular check-ups, as concluded from the proportion of children who are immunized according to immunization schedules under the age of two [18]. The study was conducted in six CCs in the city of Tampere and the town of Hämeenlinna in southern Finland. The clinics were selected on the basis of the clinics' administrative personnel's suggestion for suitable clinics. Three CCs volunteered to be intervention clinics and the remaining CCs were treated as control clinics. The study protocol was implemented during five routine visits to a public health nurse (PHN) at the CC. These visits coincided with the child's age of 2, 3, 5, 6 and 10 months.

All PHNs from the intervention clinics and the control clinics participated in the study (n = 8 and n = 6 respectively). Before the intervention began, the PHNs of the intervention clinics were trained in applying the counselling procedures, data collection and other study arrangements by the research group (12 h in total). The PHNs were also asked to practise the counselling between the training sessions with at least one client not participating in the study. The experiences were shared in small group sessions. The PHNs of the control clinics were trained for data collection and other study arrangements (6 h in total). All PHNs received a handbook in which the tasks for each research visit were explained and summarized. The researchers visited the clinics monthly during the intervention.

### Participants

**Figure 1:**
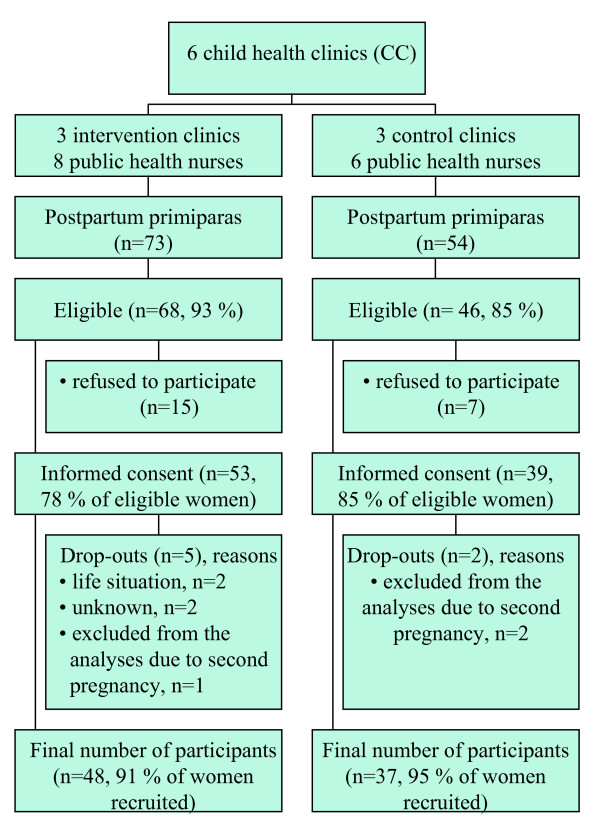
Participant flow.

All participants were primiparas. The exclusion criteria were age under 18 years, type 1 or type 2 diabetes mellitus, twin pregnancy, physical disability that prevents exercising, otherwise problematic pregnancy, substance abuse, treatment or clinical history of any psychiatric illness, inadequate language skills in Finnish and intention to change residence within three months. Between August 2004 and January 2005, the PHNs approached all postpartum primiparas in these six CCs and assessed their eligibility for the study either on their visit to the participant's home after delivery or on the first visit to the CC. All eligible women were asked to participate in the study. In total, 53 women in the intervention clinics and 39 women in the control clinics gave informed consent to participation (Figure 1). The study was approved by the Ethics Committee of the Pirkanmaa Hospital District.

### Counselling practices before the study

Information on all PHNs' usual counselling practices was collected by a questionnaire before the PHNs were trained for the study. The responses showed that the counselling practices varied widely between PHNs, but not between the PHNs of the intervention and the control clinics. However, the mean durations of time spent on counselling were short (approximately 4 min for both physical activity and dietary counselling), suggesting that the PHNs merely gave general advice on diet and physical activity rather than implementing actual counselling. During this study, the PHNs of the control clinics continued their usual physical activity and dietary counselling practices.

### Intervention clinics

#### Discussion on weight development

The PHNs had brief discussions with the participants about pre-pregnancy body weight at the child's 2-month visit to the CC. If the pre-pregnancy weight was lower than the current weight, the PHN encouraged the participant to try to return to that weight with the help of dietary and physical activity objectives (see below) during the study period. Extensive weight loss programmes, however, were not recommended.

#### Physical activity counselling

The physical activity counselling consisted of one primary counselling session (allocated time 20–30 min) at the 2-month visit and four booster sessions (allocated time 10–15 min) at the 3, 5, 6 and 10 month visits. The counselling was implemented using the model of Laitakari and Asikainen [19], which is based on two behavioural models, PRECEDE-PROCEED [20] and Stages of Change [21]. The PHN proceeded in the counselling by following a counselling card, which was filled in for each participant at each session. The primary counselling session began with a discussion about the participant's current LTPA and continued with a discussion about the participant's needs and opportunities to increase LTPA. The general benefits and restrictions of LTPA were also raised with the help of a take home leaflet. Finally, an individual weekly LTPA plan was written into the participant's follow-up notebook.

According to the physical activity recommendations for health [22] and fitness [23], which also apply to postpartum women [24,25], a minimum of 30 min of moderate-intensity physical activity on five weekdays was considered sufficient for health and a minimum of 40 min of high-intensity physical activity three times per week for fitness. By using multiples of resting metabolic equivalents (METs) with MET value 5 for moderate-intensity and MET value 7 for high-intensity LTPA [26], 800 MET minutes (METmin) was estimated to represent the minimum LTPA requirements. After making the weekly plan, the fulfilment of at least 800 METmin in the LTPA plan was checked by the PHN by multiplying the frequency, duration (minutes) and MET value of weekly LTPA. As opposed to physical activity recommendations, light-intensity LTPA (MET value 3) could also be included in the plan to improve compliance with the plan. At the booster sessions the participant's adherence to the plan was assessed, the plan was revised, if needed, and the METmin were checked.

As a part of the LTPA plan, the participant had an option to attend supervised group exercise sessions held once a week for 45–60 min at a location close to each intervention clinic. The group exercise included both endurance and muscular training and it was developed specifically for postpartum women.

#### Dietary counselling

Based on dietary recommendations [27,28], a summary of the evidence for prevention of excessive weight gain and obesity [29] and information on the diet of Finnish women [30], the dietary counselling focused on four topics that could help the participants to return to their pre-pregnancy weight. The following dietary objectives were set for each participant to achieve or to maintain: 1) to have a regular meal pattern, emphasising the importance of breakfast and ≥1 hot meal every day, 2) to eat at least 5 portions/d (400 g/d) in total of different kinds of vegetables, fruit and berries, 3) to consume mostly high-fibre bread (≥5 g fibre/100 g) and 4) to restrict the intake of high-sugar snacks to ≤1 portion/d (e.g. 50 g sweets, one pastry, once piece of cake, 2 biscuits, 2 dl ice cream or a glass of soft drink).

The dietary counselling consisted of one primary counselling session (allocated time 20–30 min) at the 3-month visit and three booster sessions (allocated time 10 min, in addition to the physical activity boosters) at the 5, 6 and 10 month visits. The model of Laitakari and Asikainen [19] was also applied to the dietary counselling. A counselling card was also used in the dietary counselling. At the beginning of the primary counselling session, the PHN assessed the participant's current dietary habits concerning these four topics using the baseline food frequency questionnaire. After comparing the personal habits to the recommendations, the PHN and the participant discussed the participant's need for dietary changes, as well as her opportunities for and barriers to making the changes. The participant also received two take home leaflets on healthy diet. The participant was asked to keep a weekly record of her compliance with the four objectives in her follow-up notebook. At each booster visit, the follow-up notebook was checked and the compliance was discussed.

### Main outcomes

The main outcome for postpartum weight retention was the proportion of women returning to their pre-pregnancy weight (weight retention ≤0 kg) by 10 months postpartum. The dietary outcomes were changes in meal pattern (breakfast and ≥1 hot meal/d), overall intake of vegetables, fruit and berries (portions/d), use of high-fibre bread (% of bread with ≥5 g fibre/100 g of total weekly amount of bread) and intake of high-sugar snacks (portions/d). The physical activity outcome was the change in the weekly METmin of LTPA.

### Data collection

Body weight was measured in light clothing and without shoes at every CC visit related to the study. The scales were calibrated to the reference scale within ± 0.5 kg at the beginning and at the end of the study. Additionally, waist circumference was measured at these visits. Data on gestational weight development was obtained from the maternity card. Pre-pregnancy weight and height were self-reported.

The baseline questionnaire including questions on background (e.g. education, smoking), dietary intake and LTPA was completed before the child's 2-month visit. The first LTPA and the dietary follow-up questionnaires were completed at the 5-month visit and the second follow-up questionnaires at the 10-month visit. These questionnaires were returned to the PHN, who checked that they were properly filled in. Information on dietary intake was obtained using a 57-item food frequency questionnaire that was a simplified version of the food frequency questionnaire used in the Health 2000 study in Finland [31]. The baseline and the follow-up dietary information were based on diet during the previous month. The questions on LTPA were modified from the International Physical Activity Questionnaire, IPAQ [32], by using the amount of breathlessness (none, some, marked) to describe light, moderate and high-intensity LTPA to the respondents. LTPA at baseline illustrated a typical week before pregnancy and at follow-up a typical week during the past three weeks. These dietary and LTPA questions have not been validated among postpartum women, however.

### Statistical methods

To test the baseline differences in background characteristics (Table 1), t-test was used for continuous variables and χ^2^-test for categorised variables. Differences in the duration of exclusive and partial breastfeeding were tested using non-parametric Mann-Whitney U test, since these variables were not normally distributed. As there were missing values in the duration of breastfeeding for 11 women, an indicator variable (0 = non-missing, 1 = missing) together with the continuous breastfeeding variables was used in the multivariable analyses to prevent the loss of data. These background variables were used, when necessary, as covariates in the multivariable analyses regardless the statistical significance of the baseline differences.

**Table 1:** Background characteristics of the participants, means (SD) or numbers (%)

	Intervention group (n = 48)	Control group (n = 37)	p-value
Age at 2 months postpartum (y)	29.5 (3.9)	28.3 (4.4)	0.21
Education level, n (%)			0.46
basic or secondary education	23 (48)	17 (46)	
polytechnic education	8 (17)	10 (27)	
University education	17 (35)	10 (27)	
Smoking status^1^, n (%)			0.22
Non-smoker before and after pregnancy	30 (68)	20 (57)	
Smoker before pregnancy and non-smoker after pregnancy	5 (11)	2 (6)	
Smoker before and after pregnancy	9 (21)	13 (37)	
Pre-pregnancy BMI (kg/m^2^)	22.7 (3.7)	22.1 (2.3)	0.36
Total gestational weight gain (kg)	16.2 (5.0)	15.3 (5.0)	0.41
Gestational weight gain, n (%)			0.78
Below recommendations^37^	9 (19)	9 (24)	
Within recommendations	13 (28)	11 (30)	
Exceeding recommendations	25 (53)	17 (46)	
Body weight at 2 months postpartum (kg)	67.1 (11.1)	64.7 (7.8)	0.26
Weight retention at 2 months postpartum compared to pre-pregnancy weight (kg)	4.3 (4.0)	4.2 (3.9)	0.91
BMI at 2 months postpartum (kg/m^2^)	24.3 (3.8)	23.6 (2.5)	0.30
by BMI groups, n (%)			0.25
18.5–24.9 kg/m^2^	30 (63)	27 (73)	
25.0–29.9 kg/m^2^	15 (31)	10 (27)	
≥ 30.0 kg/m^2^	3 (6)	0 (0)	
Waist circumference at 2 months postpartum (cm)	81.8 (9.0)	81.1 (6.7)	0.66

^1 ^Smokers include both daily and occasional smokers. Smoking status was assessed for the periods of 0–6 months before pregnancy and 4–10 months postpartum

The unadjusted differences between the groups in the proportions of women who returned to their pre-pregnancy weight were tested by χ^2^-test. The confounder-adjusted analysis of the proportions of women returning to pre-pregnancy weight, and retaining a maximum of 2 or 5 kg were done by using a logistic regression model. Analysis of covariance (ANCOVA) with confounding variables as covariates was used to test the between-group differences in average weight retention and waist circumference at 10 months postpartum, also changes in the dietary outcomes from 2 to 5 and to 10 months postpartum. As the weekly METmin were not normally distributed, they were converted into logarithms. The between-group differences of the log-transformed METmin variable at 5 and 10 months postpartum were analysed using ANCOVA of repeated measures. All statistical tests were two-sided and p < 0.05 was used as the level of statistical significance.

## Results

Figure 1 shows the flow of participants. The participants who dropped out of the study (n = 7) were younger, less educated and had higher pre-pregnancy and postpartum BMI, but lower gestational weight gain and weight retention at 2 months postpartum on average than participants who completed the study (n = 85). No major differences were observed in smoking status or in the main dietary and physical activity outcomes between the groups. There is no follow-up information available on the drop-outs.

In the intervention group, 43 (90%) women participated in all physical activity counselling sessions and 45 (94%) women in all dietary counselling sessions. All 48 women participated in the primary physical activity and dietary counselling sessions. Five women missed one physical activity booster session, three women missed one dietary booster session and three women missed the discussion about returning to pre-pregnancy weight. On average, the women participated in 4.9 of the five physical activity counselling sessions and in 3.9 of the four dietary counselling sessions. The average participation rate in the group exercise sessions was 50.7 % (sd 28.5) of the sessions available for each woman.

The differences in the background characteristics were not statistically significant between the groups (Table 1). There were also no statistically significant differences in the duration of exclusive (medians 5.0 vs. 5.0 months, p = 0.57) or partial breastfeeding (medians 10.0 vs. 8.5 months, p = 0.07) between the intervention and the control groups.

### Weight retention

**Figure 2:**
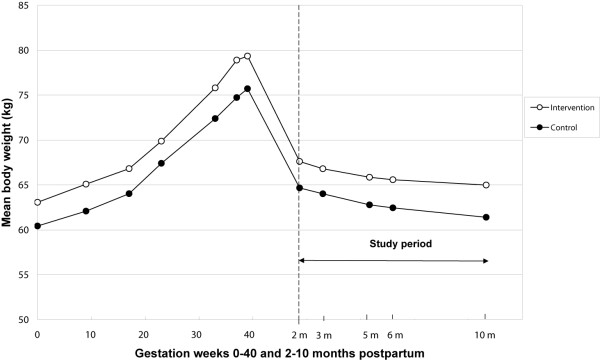
**Mean body weight changes from the beginning of pregnancy to 10 months postpartum**. Values represent unadjusted means in the intervention and the control groups. Intervention group: n = 46, except for pre-pregnancy weight (n = 45), 17^th ^gestation week (n = 42) and 37^th ^gestation week (n = 44). Control group: n = 37, except for 9^th ^and 33^rd ^gestation week (n = 36).

Figure 2 shows the unadjusted mean body weight changes during pregnancy and during the intervention (2 to 10 months postpartum) in the intervention and the control groups. Fifty percent of the intervention group and 30% of the control group returned to their pre-pregnancy weight by 10 months postpartum, but the difference did not reach statistical significance (Table 2). The confounder-adjusted odds ratio (OR) for returning to pre-pregnancy weight was 3.89 (95% CI 1.16–13.04) for the intervention group compared to the control group. The results were essentially the same when adjusted for the duration of partial breastfeeding instead of the duration of exclusive breastfeeding. The ORs for retaining maximum 2 kg or 5 kg at 10 months postpartum did not differ statistically significantly between the groups. Among those women who did not return to their pre-pregnancy weight, the unadjusted average weight retention at 10 months postpartum was 5.2 kg in the intervention group (n = 23) and 3.2 kg in the control group (n = 26). However, of these women, the intervention group had higher weight retention than the control group (6.7 vs. 5.7 kg) already at 2 months postpartum when the intervention began. Among all women, there were no differences between the groups in the adjusted average weight retention at 10 months postpartum or in the adjusted change in waist circumference from 2 to 10 months postpartum (Table 2).

**Table 2:** Comparison of weight retention^1 ^and waist circumference at 10 months postpartum between the groups. The values represent numbers (%) and odds ratio (OR) (95% confidence intervals, CI) or means (SD) and mean differences (95% CI)

	Intervention group (n = 46)	Control group (n = 37)	Intervention vs. control	p-value
Proportion of women who retained ≤0 kg, n (%)	23 (50)	11 (30)		0.06^2^
			Adjusted OR = 3.89 (1.16–13.04)	0.028^3^
Weight retention, mean (SD) (kg)	1.8 (4.3)	1.0 (4.4)	Adjusted mean difference = 0.8 (-1.1–2.7)	0.42^4^
Waist circumference at 10 months postpartum, mean (SD) (cm)	78.1 (10.2)	75.4 (6.2)	Adjusted mean difference = 1.0 (0.7–2.7)	0.24^5^

^1 ^compared with pre-pregnancy weight

^2 ^two-sided χ^2^-test

^3 ^logistic regression model, odds ratio for retaining ≤0 kg adjusted for age, education, pre-pregnancy BMI, gestational weight gain, weight at 2 months postpartum (baseline), duration of exclusive breastfeeding and smoking status. Intervention group: n = 43, Control group: n = 35.

^4 ^unadjusted means for weight retention, ANCOVA: weight at 10 months postpartum as the dependent variable, mean difference adjusted for pre-pregnancy weight

^5 ^unadjusted means for waist circumference, ANCOVA: waist circumference at 10 months postpartum as the dependent variable, mean difference adjusted for weight circumference at 2 months postpartum (baseline), age, education, pre-pregnancy BMI, gestational weight gain, duration of exclusive breastfeeding and smoking status. Intervention group: n = 43, Control group: n = 35.

### Changes in diet and physical activity

The proportion of high-fibre bread of total weekly amount of bread increased in the intervention group compared to the control group when adjusted for confounders (Table 3). The mean increase in favour of the intervention group was 16% both at the first follow-up (5 months postpartum) and the second follow-up (10 months postpartum). The intake of high-sugar snacks decreased on average by 0.6 portions/d at the first follow-up in the control group compared with the intervention group, but returned to the baseline level by the second follow-up. There were no statistically significant differences in changes in the intake of vegetables, fruit and berries between the groups. Moreover, no between-group differences were observed in the proportion of women having breakfast and at least one hot meal per day. The respective proportions of women in the intervention and the control groups fulfilling this criterion were 88% and 86% at baseline, 94% and 92% at the first follow-up and 93% and 89% at the second follow-up.

**Table 3:** Diet at baseline (2 months) and at follow-up (5 and 10 months). The values represent means (SD) and adjusted group differences (95% confidence intervals, CI) at follow-up.

	n	2 months postpartum, mean (SD)	5 months postpartum, mean (SD)	Adjusted mean difference to controls^1^	p^1^	10 months postpartum, mean (SD)	Adjusted mean difference to controls^1^	p^1^
Vegetables, fruit and berries (portions/d)								
Intervention	44	2.4 (1.3)	2.6 (1.4)	+ 0.4	0.13	2.6 (1.4)	+ 0.2	0.42
Control	37	2.7 (2.0)	2.6 (1.8)	(-0.1–0.9)		2.5 (2.1)	(-0.3–0.8)	
High-fibre bread (% of total bread)								
Intervention	44	49 (29)	60 (29)	+ 16.0	0.008	65 (27)	+ 16.1	0.008
Control	37	49 (30)	45 (33)	(4.2–27.7)		52 (31)	(4.3–27.9)	
High-sugar snacks (portions/d)								
Intervention	44	1.9 (1.2)	2.2 (1.3)	+ 0.6	0.028	2.1 (1.2)	0.0	0.93
Control	37	2.0 (1.2)	1.5 (0.9)	(0.1–1.1)		2.1 (1.4)	(-0.6–0.6)	

^1 ^ANCOVA: mean group differences, adjusted for baseline intake of the outcome variable, age, education, smoking status, gestational weight gain and BMI at 2 months postpartum. Intervention group: n = 42, Control group: n = 35

The unadjusted mean weekly METmin during leisure time were 2 328 (SD 1 308) in the intervention group and 2 061 (SD 975) in the control group before pregnancy (the baseline). At 10 months postpartum the values were 1 906 (SD 970) and 2 051 (SD 1 249) respectively. There were no statistically significant differences between the groups in changes in the weekly METmin from baseline to 5 or 10 months postpartum when adjusted for baseline weekly METmin, age, education, gestational weight gain and BMI at 2 months postpartum.

## Discussion

This study aimed at reducing postpartum weight retention in primiparas by counselling them on diet and physical activity during five of the child's routine visits to a CC. We observed that a higher proportion of the women in the intervention group than in the control group returned to their pre-pregnancy weight by 10 months postpartum, when adjusted for confounders. However, among those women who did not return to their pre-pregnancy weight, the intervention group retained more weight than the control group on average. Therefore, the average weight retention was not lower in the intervention group than in the control group.

The changes in dietary habits were modest, since only the mean proportion of high-fibre bread of total weekly amount of bread increased by 15–16 %-unit in the intervention group compared to controls from baseline to 5 and 10 months postpartum. This change corresponds e.g. to replacing one slice of low-fibre bread by one slice of high-fibre bread for every sixth slice consumed. No between-group differences were found in the intake of vegetables, fruit and berries or high-sugar snacks in favour of the intervention group. As the proportion of women having breakfast and a hot meal every day was already high at baseline, there was little potential to promote these habits by counselling. The counselling did not have an effect on the total amount of LTPA, possibly at least partly due to the fairly high level of LTPA at baseline (before pregnancy) or difficulties in arranging more time for LTPA in the new life situation.

The results of this study mostly concur with the two earlier interventions aimed at reducing postpartum weight retention [12,13]. In both of these studies, the intervention group lost more weight and/or returned to their pre-pregnancy weight more often than the control group, but no between-group differences were observed in changes in energy intake or expenditure. The methods of these studies differed from our methods to some extent. In the study by Leermakers et al. [12], women (n = 90) with at least 6.8 kg weight retention were randomised at 3–12 months postpartum either to a no-treatment control group or to a six-month behavioural weight loss intervention delivered via correspondence. In the study by O'Toole et al. [13], the participants (n = 40) were overweight women, who gained at least 15 kg during pregnancy and had at least 5 kg of postpartum weight retention at the time of recruitment (6 weeks to 6 months postpartum). They were randomized to a structured or a self-directed intervention continuing up to 1 year postpartum. These studies also had smaller sample sizes and much higher drop-out rates (31% and 41% respectively) than in our study. The drop-out rate was very low (8%) in our study, which improves the internal validity of the results. The external validity was improved by a high participation rate (81%) in a highly representative sample.

However, this study primarily piloted the study protocol for a larger study, which contributes to some limitations of this study. Firstly, the CCs were not randomized, which may have increased the baseline differences between the groups. The intervention group had slightly higher mean gestational weight gain and BMI, which are risk factors for high postpartum weight retention [4-6]. Although these variables were included in the analyses as confounders, these baseline differences, although not statistically significant, may have affected the efficacy of the intervention. The small sample size was another major limitation in this study and therefore the opportunities to observe statistically significant effects of the intervention were limited. As the number of CCs was also small, the multilevel analysis could not be used in order to take the clinic-level variation into account. Any future study should be a cluster-randomized controlled trial with a larger number of clusters and participants.

It is not clear why a higher proportion in the intervention group than in the control group returned to their pre-pregnancy weight as the effects of the intervention on dietary and LTPA habits were so minor. This discrepancy could be related to difficulties in assessing one's diet and LTPA accurately or to the limitations of our questionnaires not validated among postpartum women. The LTPA questionnaire may not have been sensitive enough in measuring changes, particularly in everyday light-intensity LTPA, which contributes significantly to the total energy expenditure. In addition, the intervention group may have decreased their total energy intake as a result of the dietary counselling, but it could not be measured by the semi-quantitative food frequency questionnaire. On the other hand, neither Leermakers et al. [12] nor O'Toole et al. [13] observed between-group differences in changes in energy intake or expenditure in their studies, although the intervention had an effect on weight retention. Concerning the validity of the weight retention outcome, body weight was measured at each visit but pre-pregnancy weight was self-reported. As overweight women usually underreport their body weight more often than thinner women [33] and there were more overweight women in the intervention group than in the control group before pregnancy, it is possible that the intervention group could have had lower average weight retention than was reported. Removing the overweight women from the analyses did not change the results essentially, however.

To our knowledge, this was the first study conducted in a primary healthy care setting aiming to reduce postpartum weight retention by dietary and physical activity counselling. The PHNs implemented the five counselling sessions on the child's routine visits to the CC and therefore the participation rate at the counselling sessions was very high. The counselling focused on promoting healthy dietary and physical activity habits. Individual recommendations for energy intake and expenditure, and thereby for energy deficit (as kJ or kcal), were not applied, because it would have been too complicated, especially as the time allocated for the counselling was short. It is possible that the women would have needed even more counselling or support to improve their dietary or physical activity habits. The time span between the last two booster sessions (4 months) may have been too long to motivate the women to adhere to the dietary and LTPA plans without support from their PHN. On the other hand, increasing the number of counselling sessions may not be feasible, since the time resources of the PHNs are limited and the main focus on the visits is on the infant's health and growth. It is possible that the presence of infants interfered with the counselling.

The need for postpartum counselling and support for healthy diet and weight management has been emphasised in several papers [34-36]. In particular, women with high pre-pregnancy BMI or high postpartum weight retention could benefit from it. Another option is that the intervention would begin in early pregnancy in order to prevent excessive gestational weight gain (the primary risk factor for high postpartum weight retention) and continue during the postpartum period.

## Conclusion

Integrating individual dietary and physical activity counselling for mothers into the routine visits to CCs increased the proportion of postpartum primiparas returning to their pre-pregnancy weight, although it did not have an effect on the average weight retention. Larger randomized controlled trials are needed to show whether counselling can improve dietary and physical activity habits in postpartum women and also to confirm the results concerning the effect of counselling on reducing postpartum weight retention.

## Competing interests

The author(s) declare that they have no competing interests.

## Authors' contributions

TIK: study design, intervention protocols (especially dietary counselling), acquisition of data, analysis and interpretation of data, and preparation of manuscript.

MP: study design, intervention protocols, statistical methodology, analysis and interpretation of data, and preparation of manuscript.

MA: study design, intervention protocols (especially physical activity counselling), acquisition of data, interpretation of data, and preparation of manuscript.

MF: study design, intervention protocols, interpretation of data and preparation of manuscript.

EW: study design, interpretation of data, and preparation of manuscript.

RL: principal researcher, obtained funding, study concept and design, intervention protocols, interpretation of data, and preparation of manuscript.

All authors read and approved the final manuscript.
